# HPV-associated cervicovaginal microbiome and host metabolome characteristics

**DOI:** 10.1186/s12866-024-03244-1

**Published:** 2024-03-22

**Authors:** Yao Zhang, Xu Wu, Dan Li, Rong Huang, Xiangyu Deng, Mingxing Li, Fukuan Du, Yueshui Zhao, Jing Shen, Yu Chen, Pingxiu Zhang, Congcui Hu, Zhangang Xiao, Qinglian Wen

**Affiliations:** 1 Department of Radiation Oncology, Cancer Center, West China Hospital, Sichuan University, Chengdu, Sichuan China; 2 Department of Oncology, The Affiliated Hospital, Southwest Medical University, Luzhou, Sichuan China; 3 Cell Therapy & Cell Drugs Key Laboratory of Luzhou, Department of Pharmacology, School of Pharmacy, Southwest Medical University, Luzhou, Sichuan China; 4 South Sichuan Institute of Translational Medicine, Luzhou, Sichuan China; 5 Yanyuan County Maternal and Child Health and Family Planning Service Center, Xichang, Sichuan China; 6 Yanyuan County People’s Hospital, Xichang, Sichuan China

**Keywords:** Human papillomavirus, Cervical screening, Cervicovaginal microbiota, Metabolomics

## Abstract

**Background:**

Cervicovaginal microbiome plays an important role in the persistence of HPV infection and subsequent disease development. However, cervicovaginal microbiota varied cross populations with different habits and regions. Identification of population-specific biomarkers from cervicovaginal microbiota and host metabolome axis may support early detection or surveillance of HPV-induced cervical disease at all sites. Therefore, in the present study, to identify HPV-specific biomarkers, cervicovaginal secretion and serum samples from HPV-infected patients (HPV group, *n* = 25) and normal controls (normal group, *n* = 17) in Xichang, China were collected for microbiome (16S rRNA gene sequencing) and metabolome (UHPLC-MS/MS) analysis, respectively.

**Results:**

The results showed that key altered metabolites of 9,10-DiHOME, *α*-linolenic acid, ethylparaben, glycocholic acid, pipecolic acid, and 9,12,13-trihydroxy-10(E),15(Z)-octadecadienoic acid, correlating with *Sneathia (Sneathia_amnii), Lactobacillus* (*Lactobacillus_iners), Atopobium, Mycoplasma*, and *Gardnerella*, may be potential biomarkers of HPV infection.

**Conclusion:**

The results of current study would help to reveal the association of changes in cervicovaginal microbiota and serum metabolome with HPV infections.

**Supplementary Information:**

The online version contains supplementary material available at 10.1186/s12866-024-03244-1.

## Background

Cervical cancer is one of the leading causes of cancer death in women [1]. It was reported in 2020 that the age-standardized global cervical cancer incidence was 13.3 cases per 100,000 women and the mortality rate was 7.2 deaths per 100,000 women [2]. In China, the age-standardized incidence rate in 2022 was 13.83 per 100,000, and the mortality rate was 4.54 per 100,000 [3]. Almost all cervical cancer (> 95%) are caused by persistent infections with one of the high-risk human papilloma virus (HPV) genotypes [4]. HPV is the most common sexually transmitted infection (STI) [5]. Risk factors include number of sextual partners, age at first sexual intercourse, immunosuppression (including HIV infection and immunosuppressive medications), altered vaginal microbiota and the presence of other STIs, such as herpes simplex [5, 6]. Some studies pointed out the relationship of HPV with age, gender, smoking, alcohol, and use of oral contraceptives [7]. Because of the vast public health burden of HPV-related cervical cancer in women, there is a necessity for identifying new secondary preventive interventions. Serum-based and/or non-serum-based biomarkers display an ideal modality for early detection or surveillance of HPV-induced cervical cancer at all sites.

There is growing evidence that the cervicovaginal microbiome plays an important role in the persistence of HPV infection and subsequent disease development [8–10]. Studies have shown that women with HPV infection had low *lactobacilli* abundance and high microbial diversity in their cervicovaginas [11], which were positively correlated with the severity of cervical lesions and specific HPVs [12]. It is highlighted that cervical microbiome has a great influence on the outcome of HPV infections [13]. Notably, HPV infection and the associated cervicovaginal microbiome varied significantly cross populations with different habits and regions [14]. Thus, specifically altered cervical bacteria may be potential biomarkers of HPV infection in different populations. Furthermore, previous study indicated that serum circulating HPV DNAs, as well as cytokine levels, vitamins and cofactors, and various gene polymorphisms have been explored [15]. A recent study showed that HPV infection remarkably changed vaginal metabolome regarding the biogenic amines, glutathione, and lipid metabolites [16]. Chorna et al. revealed an association of cervicovaginal microbiome and urine metabolome [17]. However, few studies have focused on serum metabolome. HPV infection may induce characteristic signature change of host small-molecular metabolites. Therefore, the aim of present study is to identify characteristic cervical microbiome and host metabolome for novel HPV-associated biomarkers.

It is acknowledged that, in resource-poor regions where show high-risk factors such as high fertility, early pregnancy, and early marriage, women are at a high risk of HPV infections. Xichang city is one of resource-poor areas in southwestern China, with a population of more than 4.8 million. In this study, the HPV-associated alteration of cervical microbiome and host metabolome in Xichang, China was identified through 16S ribosomal RNA gene (16S rRNA) sequencing and untargeted metabolomics. The results would help to reveal the association of altered cervical microbes and serum metabolites with the HPV infection.

## Materials and methods

### Subjects

Subjects who volunteered to participate in biomarker study were recruited from a cross-sectional study from 2019 to 2020 at Yanyuan County People’s Hospital and Yanyuan County Maternal and Child Health and Family Planning Service Center in Xichang City, China. All subjects (*n* = 76) underwent cervical screening via professional gynecological examination, thinprep cytology testing (TCT), HPV test, colposcopy, and biopsy according to the 2019 ASCCP management consensus guidelines [18]. During cervical screening, those who had menstruation, vaginal medication in the last week, post-total hysterectomy, and pregnancy were excluded for the test. The related information of all subjects (BMI, age, history of miscarriage, history of pregnancy, contraceptive method, history of menopause, etc.) was recorded. Women with a recent history of antibiotic use (less than 4 week), anti-infective treatment, urinary incontinence, hysterectomy, and pregnancy were excluded for biomarker study. All women were not given any HPV-related vaccines before. Initially included subjects underwent cytological test by TCT, and patients with TCT = ASC-US/HPV+, or TCT > ASC-US results were subjected for colposcopy. Cervical biopsy followed by histological examination (H&E stain) was conducted if suspicious lesions were identified under colposcopy. The results of the cervical biopsy were graded as normal, chronic cervicitis, CIN1, CIN2/3 and invasive cancer. Finally, all subjects were grouped into: Control group (HPV(-)/TCT(-)) and HPV group (HPV(+)/TCT(+)). Particularly, the HPV group contained those with chronic cervicitis (*n* = 7), CIN1 (*n* = 8), or CIN2/3 (*n* = 10). All subjects gave their informed consent for inclusion before they participated in the study. The study was performed in accordance with the Declaration of Helsinki, and was approved by the Clinical Trial Ethics Committee of Southwest Medical University Hospital (approval number: KY2019039).

Cervicovaginal secretion samples were collected from the peripheral cervical area and cervical secretions, scraped with a cervical swab, placed in cryopreservation tubes and stored in liquid nitrogen. Venous blood was collected in 2-mL sterilized tube and serum samples were obtained by centrifugation at 4°C (3000 rpm/min, 5 min). The serum was stored in liquid nitrogen before use. All collected samples were further processed and analyzed within 1 month.

### Microbiome analysis

Analysis of cervicovaginal microbiome was performed based on the short-read 16S rRNA sequencing [19, 20]. This technique offers high-throughput examination of microbial changes, which is convenient and powerful. However, application of 16S gene requires some assumptions, e.g., sequences of > 95% similarity represent the same genus, and sequences of > 97% similarity represent the same species [21]. Therefore, the resolution of 16S rRNA sequencing at species level is generally considered low [21].

#### DNA extraction

Total genome DNA from cervicovaginal secretion samples was extracted using the cetyltrimethylammonium bromide (CTAB) method [22]. DNA concentration and purity was monitored on 1% agarose gels. DNA was diluted to 1 ng/µL using sterile water.

#### High-throughput 16S rRNA sequencing

The amplification was conducted using the primers (341 F: 5-CCTAYGGGRBGCASCAG-3; 806R: 5-GGACTACNNGGGTATCTAAT-3) targeting the V3 and V4 hypervariable regions of the 16S rRNA gene. To differentiate each sequenced specimen and acquire accurate phylogenetic and taxonomic data, the gene products were obtained with forward and reverse error-correcting barcodes. The amplified PCR products were separated by electrophoresis on 2% agarose gel and purified by the GeneJETTM Gel Extraction Kit (Thermo Scientific, USA). Sequencing libraries were obtained using Ion Plus Fragment Library Kit 48 Rxns (Thermo Scientific) following manufacturer’s instructions. The quality of library was evaluated on the Qubit@ 2.0 Fluorometer (Thermo Scientific). Eventually, the library was sequenced on an Ion S5TM XL platform [23].

#### Data analysis

Quality filtering on the raw reads were conducted to acquire the high-quality clean reads based on the Cutadapt (V1.9.1) quality control protocol. Chimera sequences, which were detected by comparison of the reads with reference database (Silva database) by UCHIME algorithm, were removed. Sequence analysis was conducted using Uparse software (Uparse v7.0.1001). Sequences with high similarity (≥ 97%) were assigned to the same operational taxonomic units (OTUs). Representative sequence for each OTU was used for annotation of taxonomic information against Silva Database. MUSCLE software (Version 3.8.31) was used to study phylogenetic relationship of varied OTUs, and the difference of the dominant species in different groups. Abundance of OTU was normalized based on a standard of sequence number corresponding to the sample with the least sequences.

Alpha diversity indices (Observed species, Chao1, Shannon, Simpson, and ace) were calculated by QIIME software (Version 1.9.1). PCoA analysis was performed based on R software (Version 2.15.3). Linear discriminant analysis (LDA) with effect size (LEfSe) was applied to assess the differentially abundant taxon.

### Metabolomics analysis

#### Sample preparation

Serum sample (100 µL) and ice-cold methanol (400 µL) were vortex mixed. The samples were incubated at 4°C for 5 min, followed by centrifugation at 15,000 rpm for 5 min. An aliquot of supernatant was diluted by LC-MS grade water, which were subsequently centrifuged at 15,000 g at 4°C for 10 min. Finally, the supernatant were used for subsequent analysis. QC samples were prepared by mixing equal volume of all samples. For the blank sample, its pretreatment process was the same as experimental sample.

#### UHPLC-MS/MS analysis

The UHLC-MS/MS analysis was conducted using a Vanquish UHPLC system (Thermo Fisher) with an Orbitrap Q Exactive series mass spectrometer (Thermo Fisher). Samples were injected into an Hyperil Gold column (100 × 2.1 mm, 1.9 μm) using a 16-min linear gradient at a flow rate of 0.2 mL/min. The mobile phases for the positive ion mode were solvent A (0.1% formic acid in water) and solvent B (Methanol). The mobile phases for the negative ion mode were solvent A (5 mM ammonium acetate, pH 9.0) and B (Methanol). The elution gradient was as follows: 2% B, 1.5 min; 2-100% B, 1.5–12.0 min; 100% B, 12.0–14.0 min; 100-2% B, 14.0–14.1 min; 2% B, 14.1–17 min. The MS parameters in both positive/negative polarity mode were set as: spray voltage, 3.2 kV; capillary temperature, 320°C; sheath gas flow rate, 35 arb; aux gas flow rate, 10 arb.

#### Data analysis

The raw UHPLC-MS/MS data files were processed by the Compound Discoverer 3.1 (CD3.1, Thermo Fisher) where peak alignment, peak picking, and quantitation for each metabolite were conducted as we previously reported [24]. Normalization of peak intensities were performed according to the total spectral intensity. The normalized data was applied to calculate the molecular formula according to additive ions, molecular ions and fragments. The databases of mzCloud (https://www.mzcloud.org/), mzVault and MassList were used for acquiring the accurate results. Statistical analysis was conducted using the R software (R version R-3.4.3), Python (Python 2.7.6 version) and CentOS (CentOS release 6.6).

Identified metabolites were annotated by the KEGG, HMDB and Lipidmaps databases. The univariate analysis (t-test) was used to calculate p-value. Significantly differential metabolites were identified based on the following rule: VIP > 1 and p-value < 0.05 and fold change ≥ 1.2 or FC ≤ 0.833. Volcano plot was applied to filter metabolites of interest based on the Log2 (FC) and -log10 (p-value) of each metabolite. Partial least squares discriminant analysis (PLS-DA) plot was performed using SIMCA 13.0.

The functions of differential metabolites were investigated using the KEGG database. The metabolic pathway enrichment of differential metabolites was performed using Metaboanalyst (https://www.metaboanalyst.ca/). The abundance of metabolites was normalized by Autoscaling. Significantly enriched pathways were indicated with adjusted p value < 0.05 and at least 2 annotated metabolites. Biomarker models in Metaboanalyst were applied according to the PLS-DA multivariate algorithm to identify specific metabolites. The area under the receiver operating characteristic (ROC) curve was generated and the area under the ROC curve (AUC) was calculated. The cut-off of AUC for candidate biomarker was set at 0.6.

### Statistical analysis

Statistical analysis was performed with GraphPad Prism 6.0 (U.S.), and significant differences in clinical characteristics were assessed with unpaired t-test or Fisher’s exact test. Pearson correlation analysis was used to assess the relationship between metabolites or between species and metabolites. All results were considered statistically significant at *p* < 0.05.

## Results

Seventy-six participants were initially included in the microbiome metabolomics study, with 23 of them excluded from the microbiome analysis (12 sampling failures, 9 non-detects and 2 outliers) and 11 excluded from the metabolome analysis due to abnormal values. Paired microbiome and metabolomics samples from the remaining 42 participants (17 in the normal group and 25 in the HPV group) were subjected to microbiome and metabolome analyses. All HPV(+) subjects had infected one of 14 high-risk HPV genotypes: 16, 18, 31, 33, 35, 39, 45, 51, 52, 56, 58, 59, 66 and 68 (Supplementary Table 1). Seven of them had mixed infection of both low-risk and high-risk HPV (Supplementary Table 1). Analysis of clinical data from the 42 participants showed no statistical differences between the normal and HPV groups prior to collection (Supplementary Table 1), indicating that no knowable influencing factors affecting the analysis of the multi-omics study between the two groups were identified.

### Cervicovaginal microbiome structure analysis

**Fig. 1:**
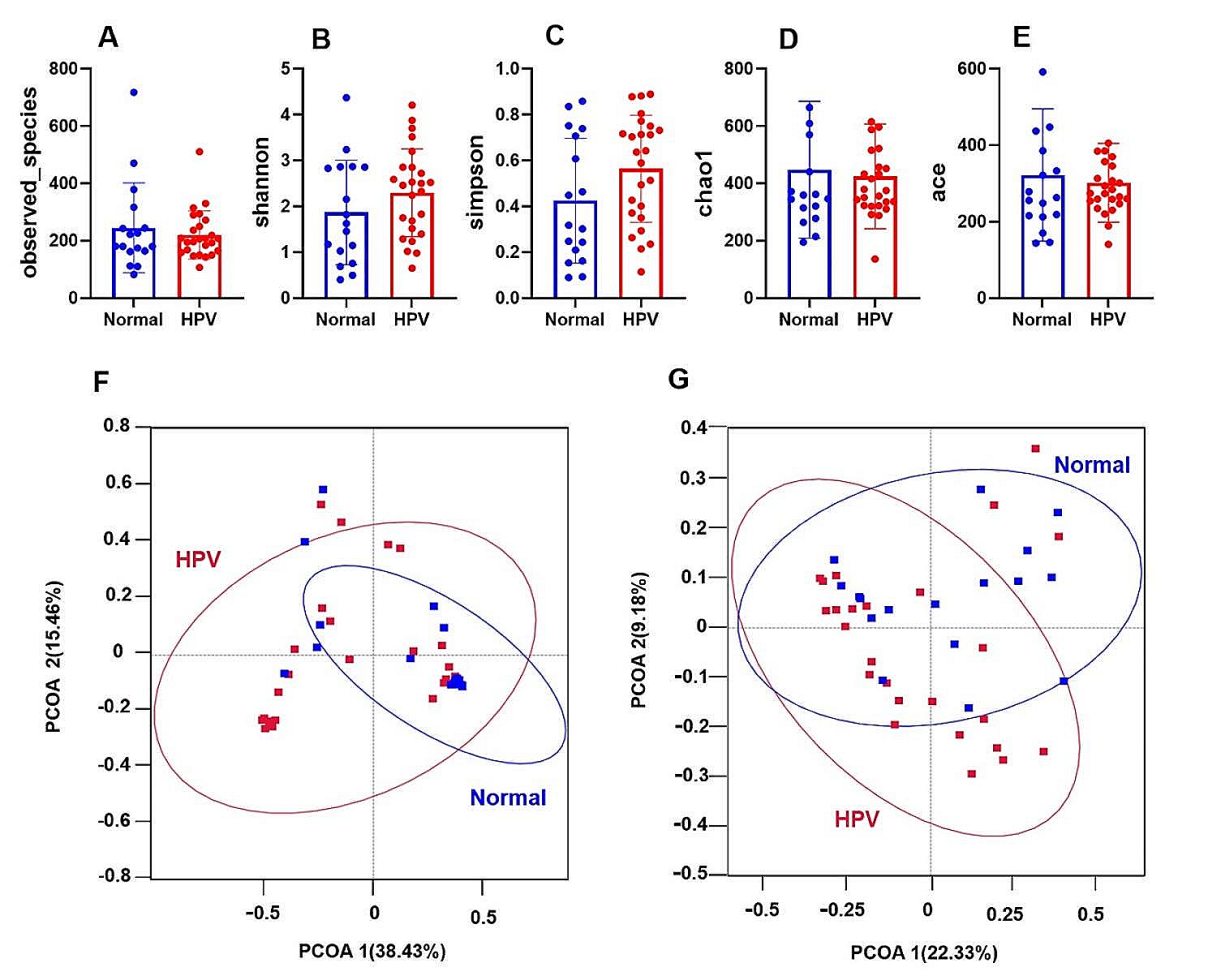
Analysis of microbial diversity in cervix and vagina. **A**–**E** Species diversity difference between normal group and HPV group was estimated by Observed-species, Shannon, Simpson, Chao1 and Ace indices. HPV (*n* = 25), group with HPV infected patients; Normal (*n* = 17), subjects with normal cervical condition. **F**–**G** unweighted and weighted PCoA plot based on OTU levels showing bacterial structure clustering. HPV group (red), Normal group (blue)

After clustering the sequences into OTUs with 97% consistency by default, 625 OTUs were obtained (Supplementary Table 2). To assess the microbial structures, the alpha diversity (Observed species, Shannon, Simpson, Chao1 and Ace, Fig. 1A and E) and beta diversity (PCoA analysis; Fig. 1F and G) of the cervicovaginal microbiota was analyzed cross groups. Although we observed a slight increase of Shannon and Simpson indices in HPV group compared to the normal group, they together with other indices (Observed species, Chao1 and ACE) did not show statistical differences. Therefore, the alpha diversity was not statistically significant between the two groups. Based on the unweighted PCoA (Fig. 1F) and weighted PCoA (Fig. 1G) plots at OTU level, the bacterial structures in the two groups had some differences yet mostly were similar. The HPV group and normal group samples were not clearly separated (Fig. 1). These results suggest that HPV infection did not significantly alter the alpha diversity of cervicovaginal microbiota and may have an impact on specific microbial structures.

### Changes in the composition of the cervicovaginal microbiota associated with HPV infection

**Fig. 2:**
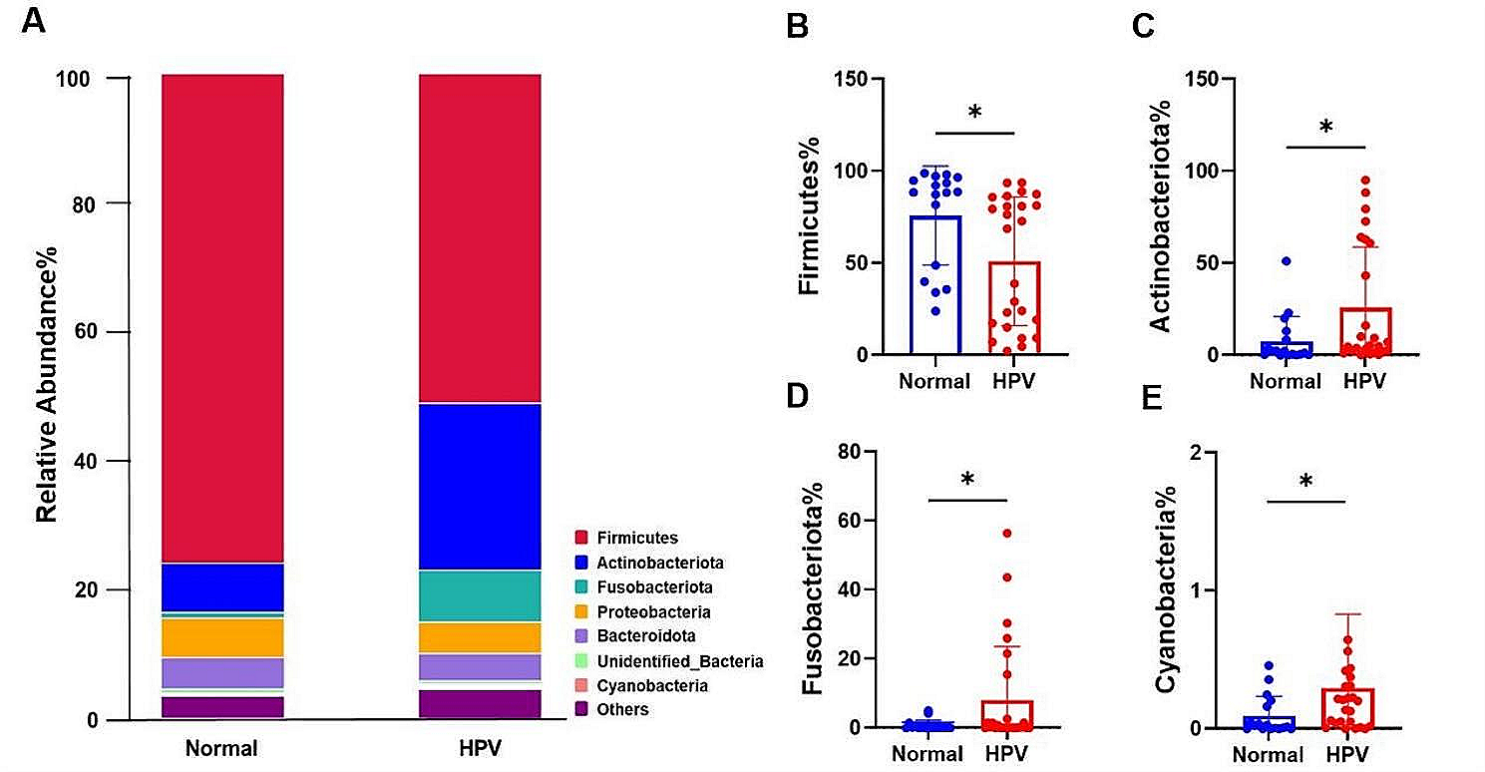
**A** Relative abundance of the most abundant 10 phylum in the HPV group and Normal group. **B**–**E** statistically comparison of Firmicutes, Actinobacteriota, Fusobacteriota and Cyanobacteria. *p < 0.05

Analysis of the top 10 species at the phylum level in the HPV and normal groups revealed significant differences in the cervicovaginal bacteria (Fig. 2). Firmicutes was the most dominant phylum, accounting for 36.07% of the HPV group and 28.03% of the normal group, respectively (Fig. 2A). The HPV group had higher levels of Actinobacteriota (18.17% vs. 2.84%), Fusobacteriota (5.70% vs. 0.26%), Cyanobacteria (0.21% vs. 0.26%) and Caldatribacteriota (0.038% vs. 0.004%) than the normal group. It is indicated that HPV remarkably impacts on cervicovaginal microbial abundance.

**Fig. 3:**
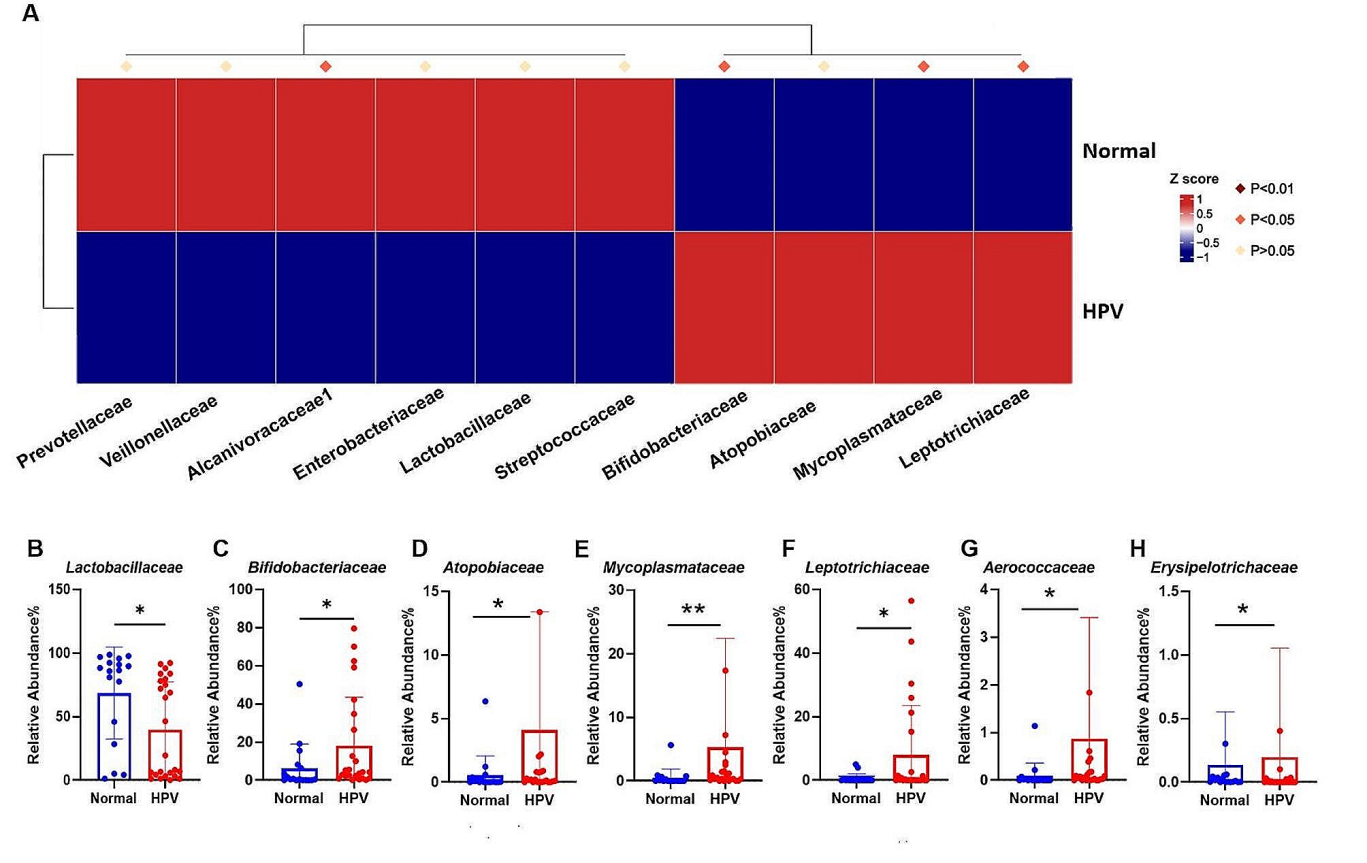
**A** Comparison of bacterial difference at family level. **B**–**H** the relative abundance of Lactobacillaceae, Bifidobacteriaceae, Atopobiaceae, Mycoplasmaceae, Leptotrichiaceae, Aerococcaceae and Erysipelotrichaceae. *p < 0.05, **p < 0.01

Welch’s t-test and Mann-Whitney test were performed on the phylum, family, genus and species level to compare the differences in cervicovaginal microbiota between the normal and HPV groups. At the phylum level, the Firmicutes (Fig. 2B) (*p* = 0.0134) was higher in the normal group than in the HPV group. The HPV group had higher numbers of the Actinobacteriota (Fig. 2C) (*p* = 0.0194), Fusobacteriota (Fig. 2D) (*p* = 0.0263), and Cyanobacteria (Fig. 2E) (*p* = 0.0231) were also significantly enriched compared to the normal group. Although the upregulation of fecal Firmicutes/Bacteroidetes ratio is considered an indicator of several pathological conditions [16], our results were the opposite (12.37% vs. 15.51%) in cervicovaginal condition. At the family level (Fig. 3A), *Lactobacillaceae* (Fig. 3B) (*p* = 0.0172) was more prevalent in the normal group than in the HPV group. Compared to the normal group, *Bifidobacteriaceae* (Fig. 3C) (*p* = 0.0194) *Atopobiaceae* (Fig. 3D) (*p* = 0.0466), *Mycoplasmaceae* (Fig. 3E) (*p* = 0.0014), *Leptotrichiaceae* (Fig. 3F) (*p* = 0.0254), *Aerococcaceae* (Fig. 3G) (*p* = 0.0169) and *Erysipelotrichaceae* (Fig. 3H) (*p* = 0.0462) were enriched in the HPV group.

**Fig. 4:**
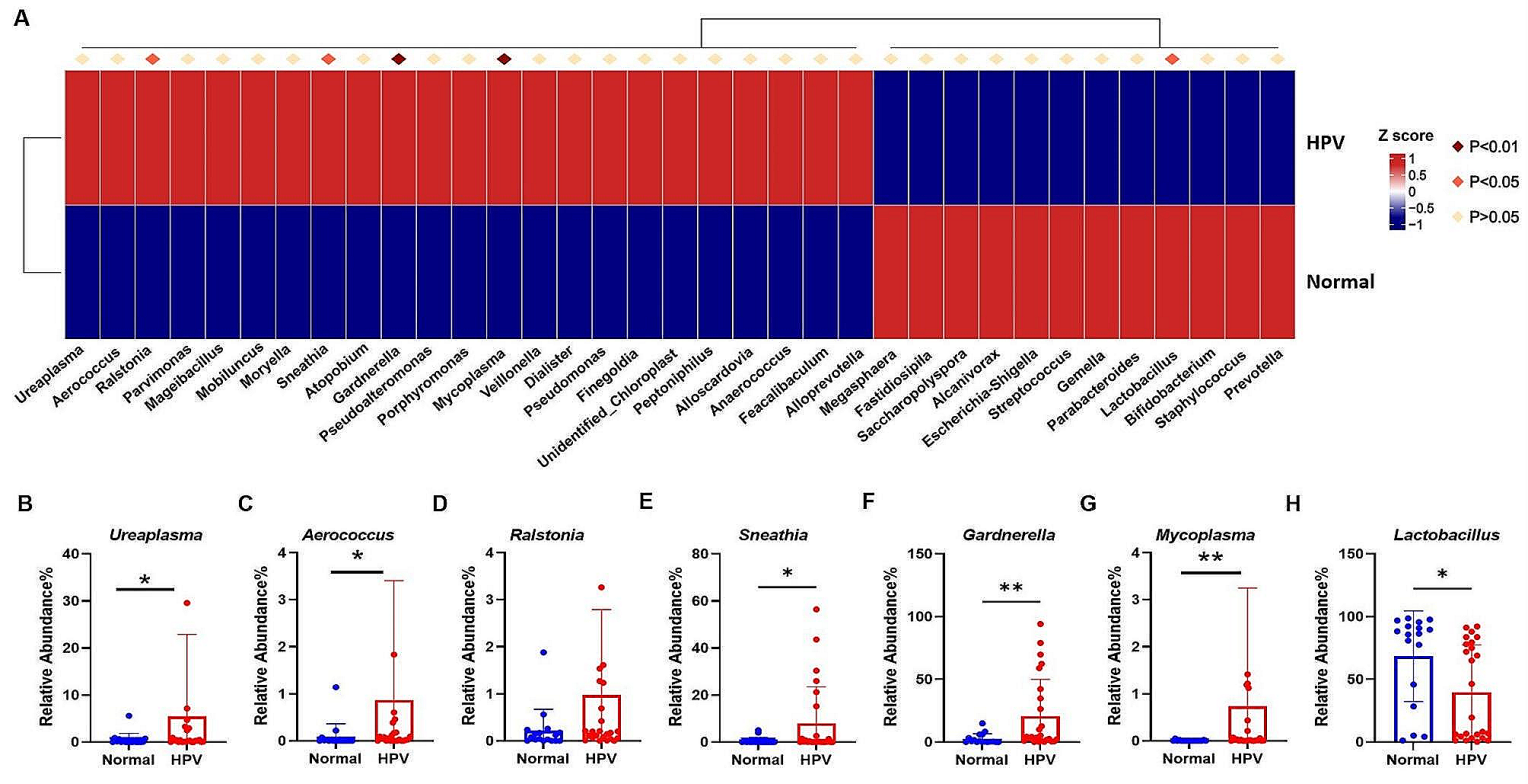
**A** Comparison of bacterial difference at genus level. **B**–**H** the relative abundance of *Ureaplasma, Aerococcus, Ralstonia, Sneathia, Gardnerella, Mycoplasma, Lactobacillus*. *p < 0.05, **p < 0.01

**Fig. 5:**
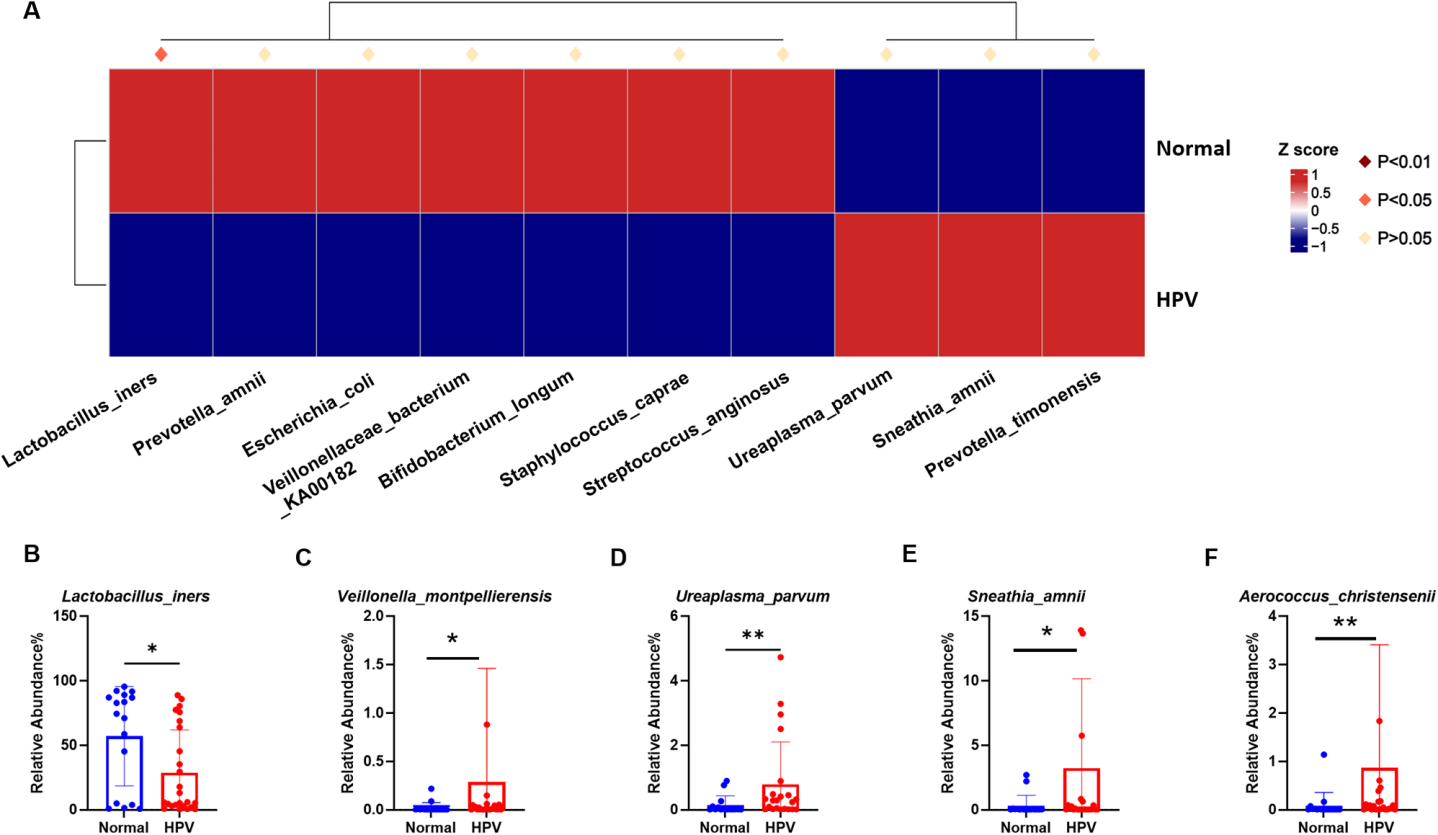
**A** Comparison of bacterial difference at species level. **B**–**H** the relative abundance of *Lactobacillus_iners, Veillonella_montpellie, Ureaplasma_parvum, Sneathia_amnii* and *Aerococcus_christensenii*. *p < 0.05, **p < 0.01

At the genus level (Fig. 4A), compared to the normal group, the HPV group had significantly higher levels of *Ureaplasma* (Fig. 4B) (*p* = 0.0106), *Aerococcus* (Fig. 4C) (*p* = 0.0105), *Sneathia* (Fig. 4E) (*p* = 0.0253), *Gardnerella* (Fig. 4F) (*p* = 0.0050), *Mycoplasma* (Fig. 4G) (*p* = 0.0021). There were differences in *Ralstonia* between the two groups, but they were not statistically significant (Fig. 4D). The normal group had significantly more *Lactobacillus* (Fig. 4H) (*p* = 0.0172) than the HPV group. At the species level (Fig. 5A), *Lactobacillus_iners* (Fig. 5B) (*p* = 0.0196) in the normal group was also significantly higher than that in the HPV group. Compared to the normal group, the HPV groups had higher abundance of *Veillonella_montpellie* (Fig. 5C) (*p* = 0.0186), *Ureaplasma_parvum* (Fig. 5D) (*p* = 0.0349), *Sneathia_amnii* (Fig. 5E) (*p* = 0.0480), and *Aerococcus_christensenii* (Fig. 5F) (*p* = 0.0096).

**Fig. 6:**
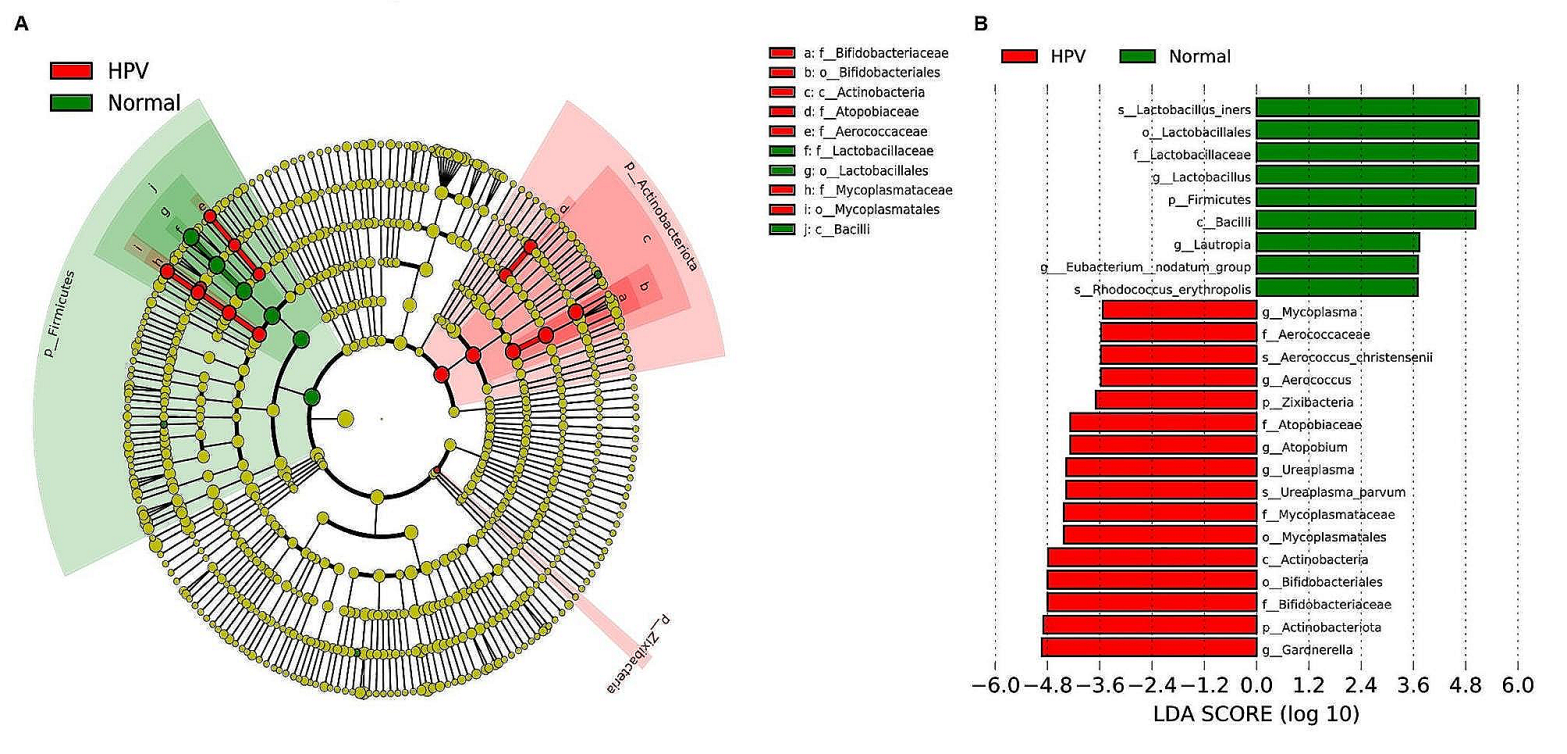
Linear discriminant analysis (LDA) integrated with effect size (LEfSe) analysis. **A** Cladogram indicating the phylogenetic distribution of microbiota correlated with the Normal or HPV groups. **B** The differences in abundance between the Normal and HPV groups

The LEfSe model was then used to identify specific microbiota that may be significantly associated with HPV infection (Fig. 6). The following bacteria were significantly enriched in the normal group (LDA score > 4.8): phylum of Firmicutes; family of *Lactobacillaceae*; genus of *Lactobacillus*; and species of *Lactobacillus_iners*. We found that the following cervicovaginal microbiota were enriched in the HPV group (LDA score > 3.6): phyla of Actinobacteriota and Zixibacteria; family of *Bifidobacteriaceae, Mycoplasmataceae*, and *Atopobiaceae*; genus of *Gardnerella, Ureaplasma* and *Atopobium*; and species of *Ureaplasma_parvum.*

### Overall overview of serum metabolites in the normal and HPV groups

A growing number of studies have found that cervicovaginal microbes are closely associated with HPV infection, and the development of cervical cancer [9, 25–27]. We hypothesized that changes in human metabolic pathways may be influenced by HPV. Therefore, we performed a metabolomic analysis of serum samples based on UPLC-MS untargeted metabolomics to identify potential HPV associated or microbial-related host metabolites.

**Fig. 7:**
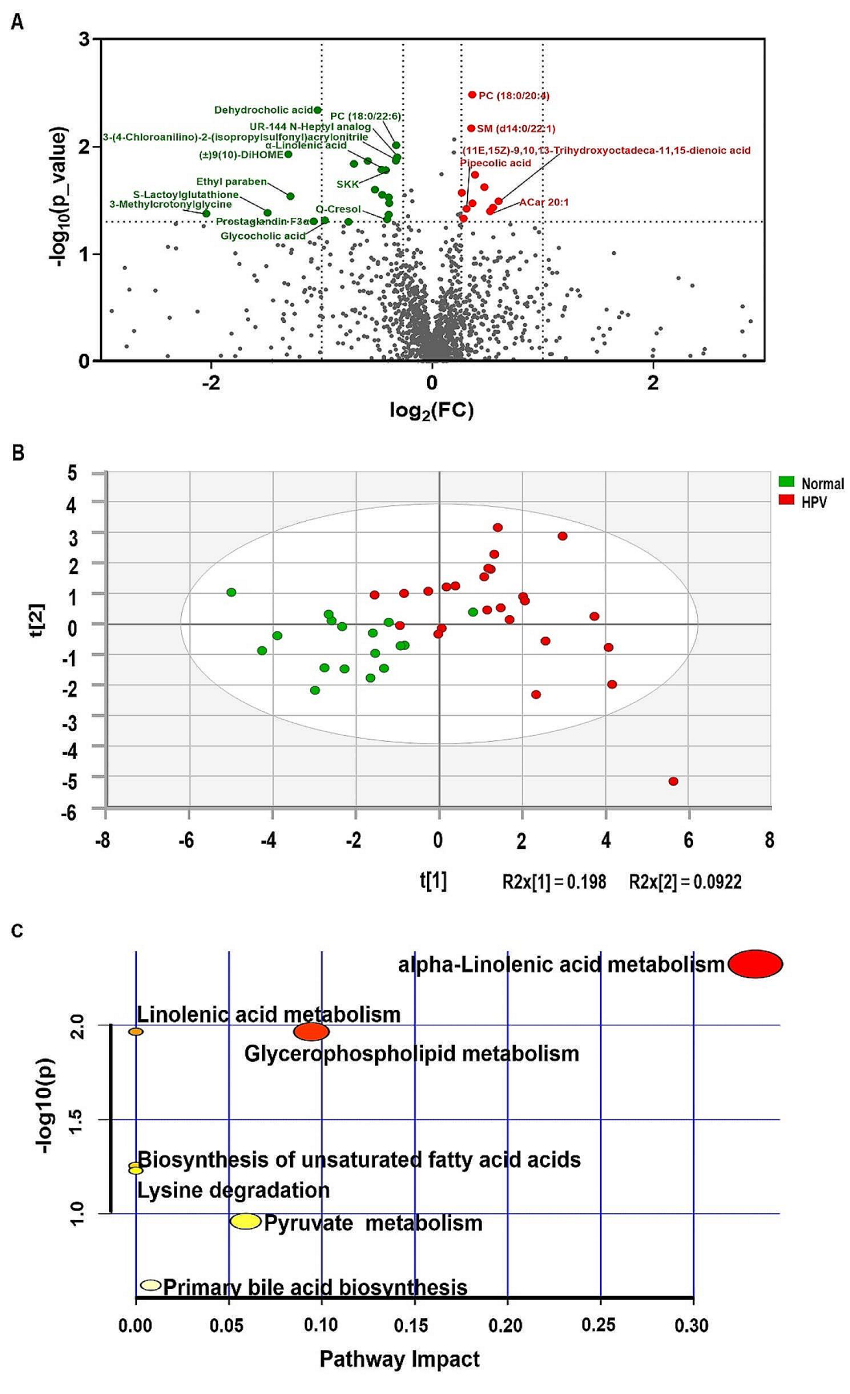
Serum metabolome analysis. **A** Volcanogram shows differential accumulation of metabolites [log2 (fold-change) on the X-axis] and significant change [-log10 (P) on the Y-axis] between the normal and HPV groups. **B** Partial least squares discriminant analysis (PLS-DA) showed differences between the normal and HPV groups. **C** bubble map showing the enriched metabolic pathway in the normal group and HPV group

We successfully identified 1488 metabolites, from both positive and negative detection mode, in the normal and HPV groups (Supplementary Table 3). A total of 32 differential metabolites were screened from the normal and HPV groups, of which 21 metabolites were down-regulated and 11 metabolites were up-regulated (Fig. 7A). Based on the relative abundance of these differentially expressed metabolites, PLS-DA was used to assess the metabolic differences between the normal and HPV groups. The results showed a significant difference in the distribution of cervicovaginal metabolites between the normal and HPV groups (Fig. 7B).

**Fig. 8:**
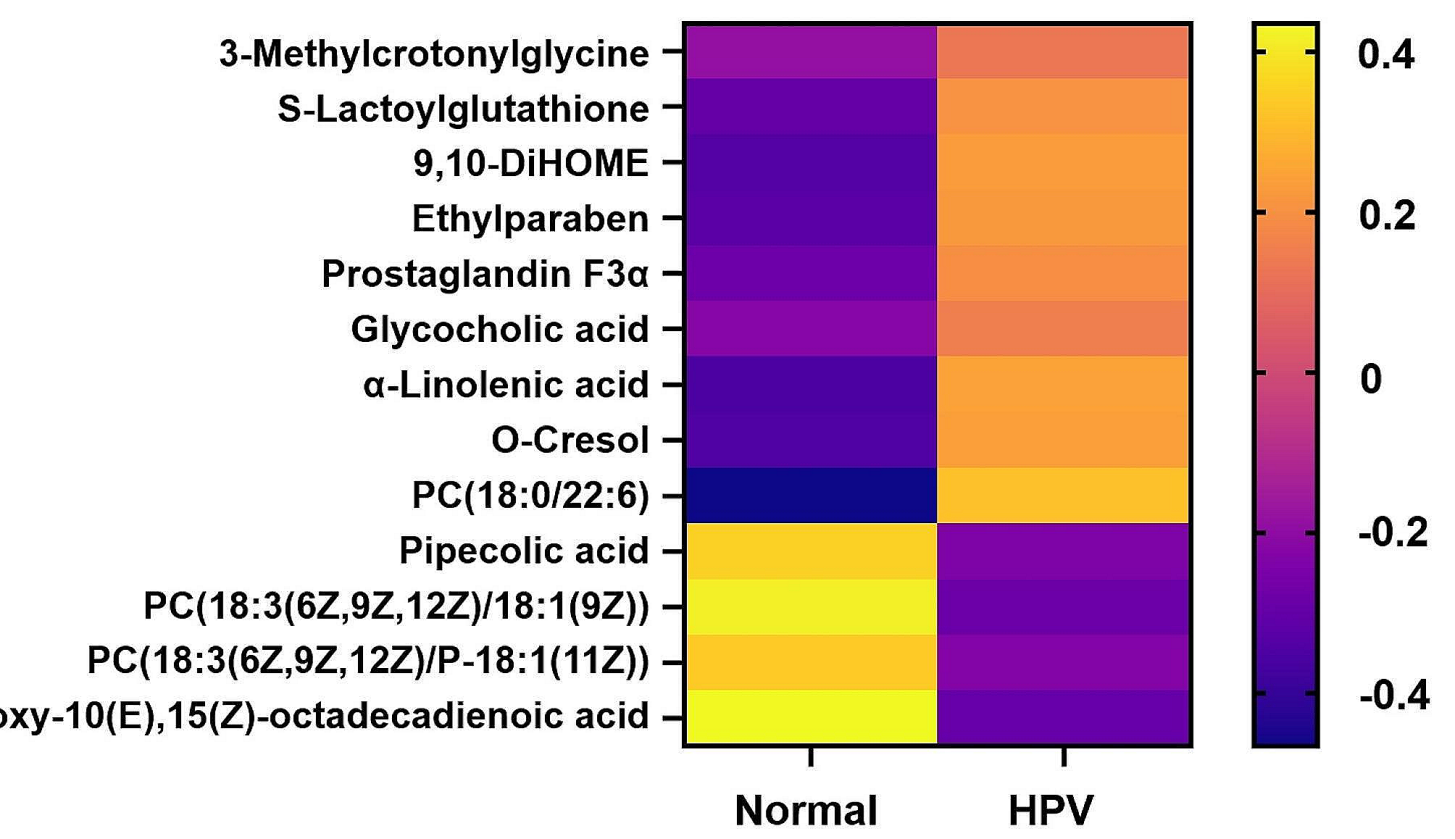
Heat maps of identified key metabolites for differentiation of normal and HPV groups

Differential metabolites were further used to predict KEGG metabolic pathways alterations (Fig. 7C and Supplementary Table 4). The identified impacted pathways included the linolenic acid metabolism, glycerol phospholipid metabolism, arachidonic acid metabolism, unsaturated fatty acid biosynthesis, lysine degradation, pyruvate metabolism, primary cholecystic acid biosynthesis. Pathway enrichment analysis based on the relative abundance of metabolites showed that the KEGG metabolic pathway of α-linolenic acid (ALA) metabolism was significantly enriched (Fig. 7C) and two metabolites (Phosphatidylcholine, (9Z,12Z,15Z)-Octadecatrienoic acid) were directly involved. Enrichment results for specific pathways are shown in Supplementary Table 5. The main enrichment pathway was related to linolenic acid metabolism. Heat map analysis showed significant differences in metabolic patterns between the normal and HPV groups based on the identified biomarkers and metabolites of the enriched pathways (Fig. 8).

### Identification of specific metabolites for HPV infection

**Fig. 9:**
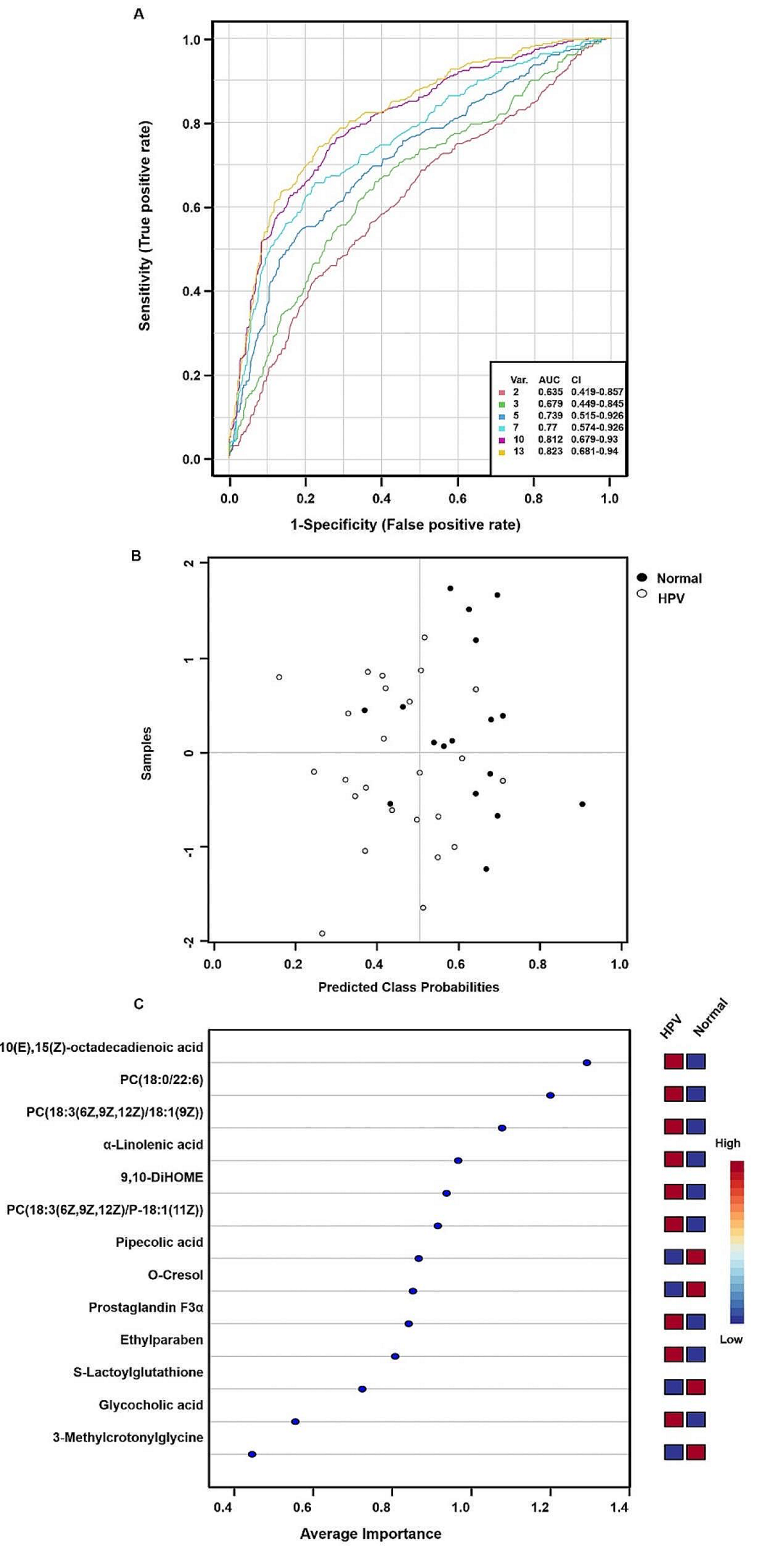
Important discriminatory metabolites identified by correlation and multivariate analysis between the Normal and HPV groups. **A** ROC curve based on cross validation (CV) performance. The variables used in the model are displayed in **C** below. **B** the predictive class probabilities for each sample based on AUC. **C** The PLS-DA model obtained significant discriminatory metabolites of average importance

To further identify metabolite changes associated with HPV infection, biomarker analysis was performed. The area under the ROC curve for the 13 variable model was 0.823 (CI: 0.689–0.94) (Fig. 9A). The predicted probability of classification for all samples indicated that the normal and HPV groups were well classified (Fig. 9B). Figure 9C shows the 13 most important biomarkers, including 9,12,13-Trihydroxy-10(E),15(Z)-octadecadienoic acid, PC (18:0/22:6), PC (18:3 (6Z,9Z,12Z) /18:1(9Z)), α-linolenic acid, 9,10-DiHOME, PC (18:3 (6Z,9Z,12Z) /P-18:1 (11Z)), pipecolic acid, O-cresol, prostaglandin F3α, ethylparaben, S-lactoylglutathione, glycocholic acid, and 3-methylcrotonylglycine. The ROC curves of these potential candidate biomarkers showed that the AUC of all candidate biomarkers was above 0.656 with a P value less than 0.05, indicating that they might be significantly associated with HPV infection (Supplementary Table 6).

### Correlation analysis reveals an overview of the microbial metabolic axis of HPV infection

Based on the microbiome and metabolomics data described above, we subsequently performed Pearson correlation analysis to identify relevant microorganisms and metabolites in the HPV and normal groups.

**Fig. 10:**
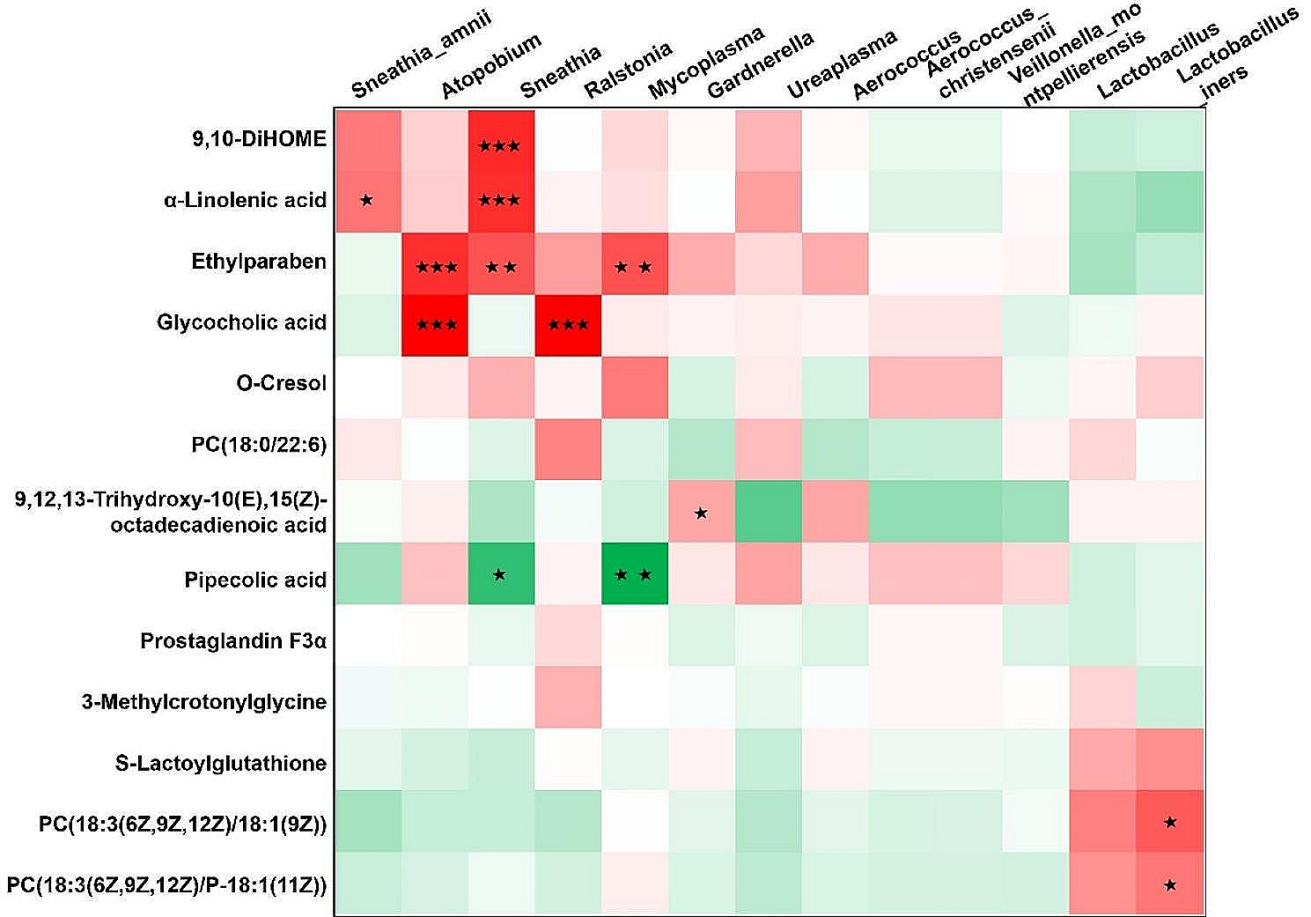
Integrated correlation-based analysis (Pearson’s correlation) of key altered microbes and metabolites upon HPV infection

As shown in Fig. 10, 9,10-diHOME was significantly positively correlated with *Sneathia* (*p* = 0.0006). The ALA was positively correlated with *Sneathia-amnii* (*p* = 0.0412) and *Sneathia* (*p* = 0.0008), but was significantly negatively correlated with *Lactobacillus* and *Lactobacillus_iners*. *Ethylparaben* was positively correlated with *Atopobium* (*p* = 0.0077), *Sneathia* (*p* = 0.0077) and *Mycoplasma* (*p* = 0.0069). Glycocholic acid (*p* = 0.0069) was positively correlated with *Atopobium* (*p* = 0.00002) and *Ralstonia* (*p* = 0.00001). Pipecolic acid was negatively correlated with *Sneathia* (*p* = 0.0161) and *Mycoplasma* (*p* = 0.0026). *Gardnerella* was positively correlated with 9,12,13-Trihydroxy-10(E),15(Z)-octadecadienoic acid (*p* = 0.0492). *Lactobacillus_iners* was positively correlated with PC (18: 3(6Z,9Z,12Z) / 18: 1 (9Z)) (*p* = 0.0120). The results showed that 9,10-DiHOME, ALA, ethylparaben, glycocholic acid, pipecolic acid, 9,12,13-trihydroxy-10(E),15(Z)-octadecadienoic acid, PC (18:3(6Z,9Z, 12Z) / 18:1(9Z)) and PC (18:3(6Z,9Z,12Z) / P-18: 1 (11Z)), correlating with *Sneathia (Sneathia-amnii), Lactobacillus* (*Lactobacillus_iners), Atopobium, Mycoplasma*, and *Gardnerella*, may be potential biomarkers of HPV infection.

## Discussion

HPV prevalence statistics in recent years has been reported in China, ranging from 8.42 to 21.1% [5, 28] The prevalence of HPV as well as the associated microbiome-metabolome feature in Sichuan, particularly in the poor regions, has not yet been investigated. In the present study, we investigate the HPV-associated cervicovaginal microbiome and host metabolome characteristics in western Sichuan, China.

Cervicovaginal microbial structure has been associated with HPV infection [27], high-risk HPV infection type [26], and cervical abnormalities [29, 30]. For the female reproductive tract, health is usually associated with low microbial diversity and the predominance of only one or a few lactic acid bacteria (*lactobacillus* spp., including *L. gasseri*, *L. crispatus*, *L. jensenii*, and *L. iners*) [31–33]. A decrease in *lactobacillus* and an increase in bacterial species diversity were associated with various cervical diseases such as bacterial vaginosis (BV), vulvovaginal atrophy (VVA) [34], HPV infection [35], and progression of cervical precancerous lesions [12]. Generally, normal cervicovaginal microecology dominated by *lactobacillus* helps to maintain a stable pH at 3.8–4.5, which reduces HPV infection and inhibit persistent HPV infection [36]. The acidic environment constrains the outgrowth of several opportunistically pathogenic strains, such as *Chlamydia trachomatis*, *Gardnerella vaginalis*, and *Neisseria gonorrhoeae* [37]. *Lactobacillus*-derived lactic acid also enhances the viscosity of cervicovaginal mucus, increases viral particle trapping, modulating inflammatory immune response and suppressing the growth and migration of cervical cancer cells [38]. In addition, other beneficial microbiota can also generate antimicrobial peptides of bacteriocins, which exhibits bactericidal effect and regulating inflammation [39]. Consistently, the normal group in the present study was dominated by *Lactobacillus* and the abundance of other microorganisms was low. On the contrary, HPV infection led to significantly reduced *Lactobacillus*, particularly for *Lactobacillus_iners*. Other *Lactobacillus spp.* were not significantly altered.

Studies have shown that *Atopobium* and *sialidase* genes could serve as microbial markers of persistent HPV infection [36]. The finding that *Ureaplasma parvum* infection expressed *UpVF* and induced vacuolated HeLa cell death suggested a potential pathogenic mechanism between *Ureaplasma parvum* infection and cervical carcinogenesis [40]. *Sneathia* strains was positively associated with cervical cancer [41, 42]. Particularly, *Sneathia_amnii* was reported associated with bacterial vaginosis, preeclampsia, preterm labor, spontaneous abortion, postpartum bacteremia, and other invasive infections [42]. *Mycoplasma* infection increased the prevalence of high-risk HPV infection, which may be a factor in the persistence of high-risk HPV infection that exacerbated cervical lesions [43]. *Veillonella_montpellierensis* infection may cause disease by depleting cervicovaginal lactate [44]. *Aerococcus_christensenii* caused a potentially life-threatening membrane infection in the vagina and cervix [45]. In the present study, the results showed that HPV infection resulted in increased *Ureaplasma* (*Ureaplasma_parvum*), *Aerococcus* (*Aerococcus_christensenii*), *Sneathia* (*Sneathia_amnii*), *Gardnerella*, *Mycoplasma* and *Veillonella_montpellie*. LefSe analysis showed that the phylum-level Actinobacteriota and the genus-level *Gardnerella*, *Ureaplasma* and *Atopobium* may be potential features of HPV infection, both of which have high LDA scores. These findings were generally consistent with previous documents. It is suggested that there was a complex association and vicious circle among bacterial changes, HPV infection, and cervical lesions, which together contributed to the development and progression of cervical disease initiation and progression.

Subsequently, an untargeted metabolomic analysis of serum samples from HPV-infected patients was performed, cooperating with microbial analysis to identify potential metabolic biomarkers for HPV infection. Through multiple comparison, multivariate and correlation analysis, we identified several significantly altered metabolites, including 9,10-DiHOME, ALA, ethylparaben, glycocholic acid, pipecolic acid, 9,12,13-trihydroxy-10(E),15(Z)-octadecadienoic acid, PC (18:3(6Z,9Z, 12Z) / 18:1(9Z)) and PC (18:3(6Z,9Z,12Z) / P-18: 1 (11Z)). We established a 13-variable model (containing the 13 significantly altered metabolites) to reveal their association with HPV infection. Notably, the area under the ROC curve for the 13-variable model was 0.823 (CI: 0.689–0.94), although the area under the ROC curves for all individual metabolites (≥ 0.656, *p* < 0.05) were not high. It is thus highlighted that these significantly altered metabolites were potential candidate biomarkers that were significantly associated with HPV infection and cervical bacteria alterations. Previous studies showed that ALA was found to reduce the expression of HPV tumor proteins E6 and E7 and restore the expression of tumor suppressor proteins p53 and Rb [46] and have oncogenic effects. Meanwhile, ALA suppressed cell proliferation in prostate, breast and bladder cancers [47, 48] and up-regulated tumor suppressor genes, playing an suppressive role on various tumors [49]. The correlation between placental ethylparaben and cord blood gamma-glutamate transferase (GGT) and glucose levels provides a starting point for further studies on the mechanisms of uterine metabolic processes associated with parabens [50]. Glycocholine was used as a metabolic biomarker for traumatic brain injury (TBI) via assessing the duration of TBI injury [51]. Glycocholic acid metabolism was closely associated with certain strains of *Bacteroides* [52]. Either in anaerobic or aerobic cultures *Bacteroides* are unable to convert the side chains of bile acids. (±)9(10)-DIHOME induced oxidative stress at high concentrations [53]. Through correlation analysis, we found that there was strong correlation among cervical microbiota alteration and serum metabolism during HPV infections. For instance, ALA and *Lactobacillus* were closely related in HPV infection.

Omics study has been widely applied for biomarker screening and identification in the past decade. Disease-specific biomarkers facilitate diagnosis of diseases as well as monitoring of response to therapy in individuals. However, discovering disease-specific biomarkers faces several challenges, such as determining the causal relationships of observed changes, understanding their functions in disease initiation and progression, and the individual or populational heterogeneity [54]. Therefore, the identified potential biomarkers from microbiome and metabolome analysis in the present study still require further validation in larger clinical cohort and future causality study based on the understanding of disease mechanism.

The limitation of current study included the following. Firstly, the sample size of this study was considered small. To propose the identified microbes and/or serum metabolites as biomarkers, the findings require further unbiased validations in an independent and larger sample cohort. Besides, due to the small sample size, it is unable to see the differences depending on CIN grades, HPV types and vaginal community state types (CSTs). Secondly, the current methodology did not take into consideration that whether HPV persistently or non-persistently infected the included subjects. Some of the CIN subjects with HPV infection had the chance to be recovered to normalcy due to clearance by host immunity. Thirdly, this study did not perform causality experiments to confirm whether the identified metabolic markers were specific to vaginal microbiome alteration.

## Conclusion

In this study, we showed that 9,10-DiHOME, ALA, ethylparaben, glycocholic acid, pipecolic acid, 9,12,13-trihydroxy-10(E),15(Z)-octadecadienoic acid, PC (18:3(6Z,9Z, 12Z) / 18:1(9Z)) and PC (18:3(6Z,9Z,12Z) / P-18: 1 (11Z)), correlating with *Sneathia (Sneathia-amnii), Lactobacillus* (*Lactobacillus_iners), Atopobium, Mycoplasma*, and *Gardnerella*, may be potential candidates for biomarker of HPV infection.

### Electronic supplementary material

Below is the link to the electronic supplementary material.

Supplementary Material 1

## Data Availability

The datasets generated and/or analysed during the current study are available in the NCBI Sequence Read Archive (SRA) repository (accession number, PRJNA1046567).

## Abbreviations

ALA: α-linolenic acid
AUC: area under the curve
CI: confidence interval
GM-CSF: granulocyte-macrophage colony-stimulating factor
GGT: gamma-glutamate transferase
HPV: human papillomavirus
LDA: linear discriminant analysis
PCoA: principal coordinate analysis
ROC: receiver operating characteristic
16S rRNA: 16S ribosomal ribonucleic acid
TCT: Thinprep cytology testing
VVA: vulvovaginal atrophy
